# Corrosion Behavior of Friction Stir Welded AA8090-T87 Aluminum Alloy

**DOI:** 10.3390/ma15155165

**Published:** 2022-07-26

**Authors:** Chandrasekaran Shyamlal, Rajesh Shanmugavel, J. T. Winowlin Jappes, Anish Nair, M. Ravichandran, S. Syath Abuthakeer, Chander Prakash, Saurav Dixit, N. I. Vatin

**Affiliations:** 1 Department of Mechanical Engineering, Kalasalingam Academy of Research and Education, Krishnankoil 626126, India; shyadavidhars@gmail.com (C.S.); s.rajesh@klu.ac.in (R.S.); winowlin@klu.ac.in (J.T.W.J.); anishn@live.com (A.N.); 2 Department of Mechanical Engineering, K Ramakrishnan College of Engineering, Tiruchirappalli 621112, India; smravichandran@hotmail.com; 3 Department of Mechanical Engineering, PSG College of Technology, Coimbatore 641004, India; ssa.mech@psgtech.ac.in; 4 School of Mechanical Engineering, Lovely Professional University, Phagwara 144411, India; 5 Division of Research and Development, Lovely Professional University, Phagwara 144411, India; 6 Peter the Great St. Petersburg Polytechnic University, 195251 Saint Petersburg, Russia; vatin@mail.ru; 7 Division of Research & Innovation, Uttaranchal University, Dehradun 248007, India

**Keywords:** precipitates, hardness, surface roughness, corrosion, grain boundary, pits

## Abstract

Aerospace alloys with reduced wall thickness but possessing higher hardness, good tensile strength and reasonable corrosion resistance are essential in manufacturing of structures such as fuselage. In this work, friction stir welding has been carried out on such an aerospace aluminum alloy AA8090 T87 which contains 2.3% lithium. Tool rotational speed of 900 rpm and traverse speeds of 90 mm/min., 110 mm/min. are the welding parameters. Hardness analysis, surface roughness analysis and corrosion analysis are conducted to analyze the suitability of the joint for the intended application. The samples were corrosion tested in acid alkali solution and they resulted in the formation of pits of varying levels which indicate the extent of surface degradation. Hardness of the samples was measured after corrosion analysis to observe the changes. The analysis suggests that the change in tool traverse speed transformed the corrosion behavior of the joint and affected both the hardness and surface roughness which mitigated the quality of the joint.

## 1. Introduction

The efficacy of Friction Stir Welding (FSW) technology, a solid state joining process [1] founded by TWI (The Welding Institute) [2,3,4] is used in the aerospace structures due to its production of strong metallurgical joint compared to fusion welding technology. In addition, the resulting weld joints have improved mechanical properties. The finer recrystallized equiaxed grains [5] produced by FSW technique is responsible for adequate strength, toughness and ductility. In FSW, precipitate formation, grain boundary strengthening and dislocation hindrances are the major mechanisms which lead to increased weld performance. The grain boundary strengthening is achieved through increasing the rotation speed and reducing the traverse speed. My adjusting the parameters such as rotational speed and traverse speed the metallurgical characteristics such as precipitation and grain boundary strengthening can be achieved. More the grain boundaries are created during the weld, the smaller the size of the grains become. The grain boundary density and mis-orientation created in the weld zone can be analyzed through EBSD (Electron Back Scattered Diffraction) technique [6].The sub grain boundaries formed due to rotation of the tool [7] in the stir zone (SZ) stops more dislocations by the increased grain boundary area. Hence, the combined effect of precipitate formation and equiaxed grains in the stir zone [8] enhances the joint efficiency. Thus it is understood that the precipitates and dislocations play a major role in increasing the hardness of the structure [9,10]. However, the higher rotational speed will lead to the dissolution [11,12] of precipitates in the stir zone which leads to the decrement in the hardness in the stir zone [13] but enhances the corrosion resistance.

Further, the inclusion of lithium (>2%) as an alloying element improves the elastic modulus of the material. Cu and Mg are added to aluminum lithium alloys to impart strength [14] to the alloy by forming Al-Mg-Cu, Al-Cu-Li precipitates. These precipitates maximize the properties such as hardness and tensile strength of the structure [15]. The precipitate formation may benefit the mechanical properties but it will also affect the corrosion behavior [16]. The corrosion resistance of the joint is considered as an important criterion for the evaluation of joint efficacy. Alloy manufacturing process involves the addition of different alloying elements and as these alloying elements occupy different positions in electrochemical series, they have differing reactivity with the matrix [17]. The formation of intermetallic compounds due to differing electronegativity values will create heterogeneous precipitates such as Al_2_Cu [18] in the grain boundaries [19] and in the vicinity of dislocations which may corrode during service. These type of intermetallic compounds forms at the grain boundaries predominantly than inside the grain. These intermetallics may be the reason for the formation of eutectics in the grain boundary. The indication of corrosion occurrence is pit formation [20] in the surface of the material and in grain boundaries which acts as notch and propagate the crack through it. In this work, the major components responsible for pit formation are Al_2_Cu (Al-Cu) precipitates. Further, severity in corrosion causes decrease in elongation [21,22] and increase in the surface roughness which finally causes catastrophic failure to the structure.

Limited researchers have investigated mechanical properties of friction stir welded aluminum alloys. However, the reported literatures on the corrosion perspective of AA8090 in the context of precipitate formation are very scanty. The objective of this work is to investigate the hardness, surface roughness, precipitate formation and the effect of precipitate formation on corrosion resistance of AA8090 aluminum alloy.

## 2. Materials and Methods

**Figure 1:**
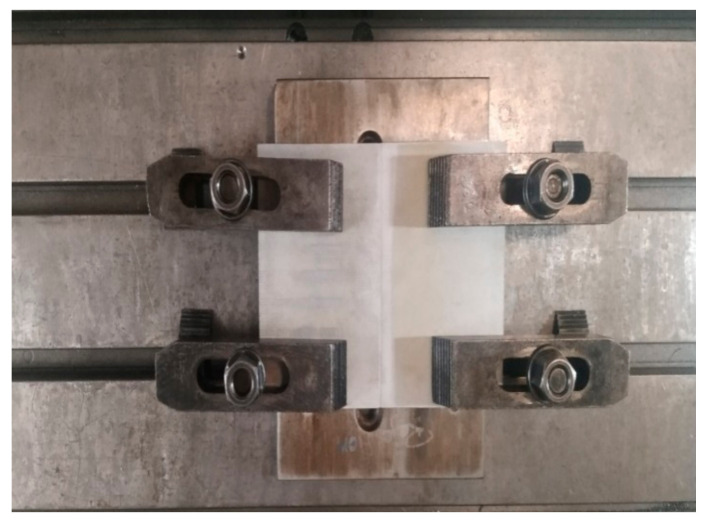
Machine set up for welding.

**Figure 2:**
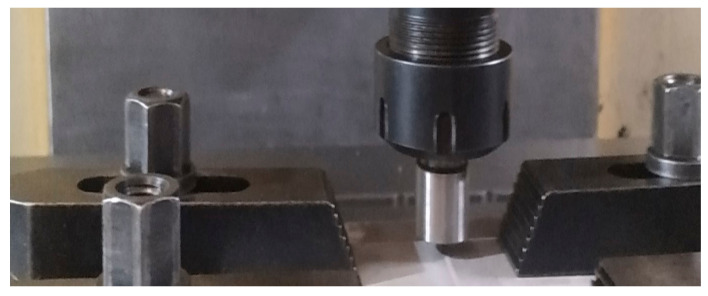
Friction stir welding tool.

The friction stir welding has been executed using CNC machining center (Make: Hurco, Indianapolis, IN, USA). The used sample size is 150 mm × 100 mm × 5 mm which is procured commercially from Bharat Aerospace Alloys, Mumbai, India. The chemical composition of the sample was verified using Optical Emission Spectroscopy (OES) [23,24]. The samples have been cut to specified size by Electronica maxi-cut wire-cut EDM machine. The tool used for welding is H13 tool steel having tool pin height of 4.7 mm, pin root diameter 5 mm and tip diameter 3 mm with shoulder diameter of 23 mm. Figure 1 and Figure 2 shows the machine set-up and the FSW tool used for welding. The joint description is provided in Table 1. The chemical composition of the tool and work is shown in Table 2 and Table 3. The hardness testing was performed with 500 gf load, 15 s dwell time using MVH-TS1 make micro-Vickers hardness machine according to ASTM standard E384 using 70 mm × 10 mm × 5 mm strip (polished work piece) [25]. The corrosion test coupons have been extracted from the welded area of size 30 mm × 30 mm × 5 mm, polished and immersion corrosion test was conducted by dipping the sample in 57 g/L NaCl (neither acidic nor alkaline) + 10 mL/L H_2_O_2_ (a weak acid) solution for 6 h at 32 °C as per ASTM standard G110 [26]. Scanning electron microscopy images were taken using Jeol 6000 plus microscope (Akishima, Japan) to investigate the sub surface after etching the sample using 85 mL distilled water, 15 mL HF, 5 mL H_2_SO_4_ [27,28,29]. Electron Back Scatter Diffraction (EBSD) analysis has been carried out to analyze the grain boundary mis-orientation.

## 3. Investigation of Mechanical Properties

### 3.1. Analysis on Hardness of the Weld

This section presents the variation of hardness measurements and the variation of hardness across the friction stir welded zone. Generally, the hardness varies based on the variation in the grain size and also with respect to the precipitate formation in the substrate (in this case aluminum) which will come into effect in the weld zones, based on the heat input in the work material.

**Figure 3:**
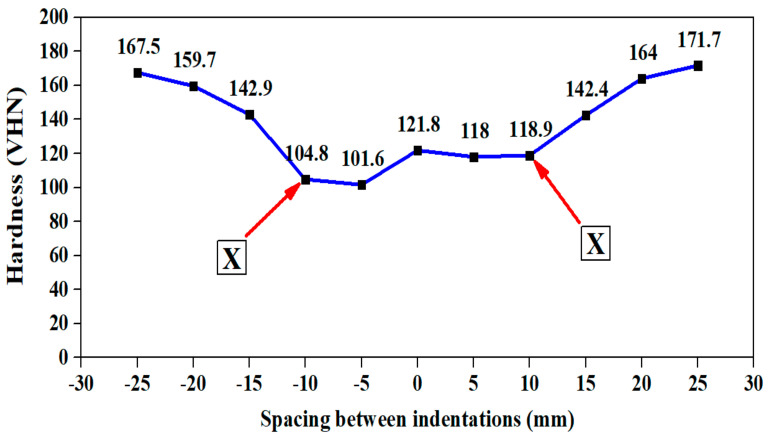
Hardness Map for 900-90 joint. X–Hardness transition point.

**Figure 4:**
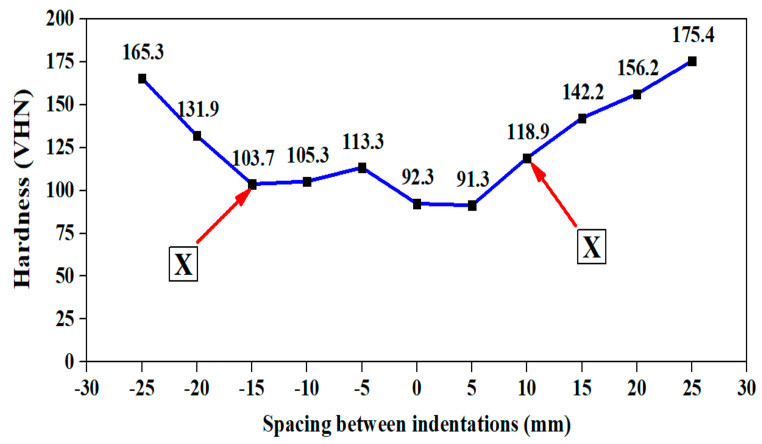
Hardness map for 900-110 joint. X–Hardness transition point.

The source of heat generation is in the stir zone. The heat developed in the stir zone was dispersed throughout the workpiece. The hardness on the base metal is escalated due to the formation of intermetallic compounds Al-Cu-Mg and Al-Cu-Li as confirmed from Table 2. Figure 3 and Figure 4 confirms that recrystallization occurs in and around the stir zone. This zone is also referred to as the Thermo Mechanical Affected Zone (TMAZ) and is characterized by presence of fine grains and coarse precipitates. More precipitates become dissolved as a solid solution in the stir zone and in TMAZ which is observed from the lesser hardness distribution region observed adjacent to the stir zone on both the joints. Secondary reason being, T87 heat treatment was removed in the stir zone due to annealing kind of treatment which took place during welding. The above figures also show the sharp decline in hardness up to point X from the cold worked state (that is from base metal hardness) on both the sides of the joint due to heat escalation. There was an asymmetrical plateau region near the Stir Zone (SZ) in the hardness map of 900-110 joint. This asymmetrical behavior in hardness is attributed to the reduction in the grain boundaries on one side of the joint. However, there is a symmetrical plateau region near the stir zone in the hardness map of 900-90 joint. This indicates the presence of higher grain boundary area and significant precipitation. Hence, the only strengthening mechanism which played crucial role in the stir zone is grain boundary strengthening mechanism along with precipitate formation. The hardness on the advancing side is lesser due to severe plasticization by the tool in the weld region on both 900-90 joint and 900-110 joints. The common observation from both the hardness maps is that the hardness varied with respect to the distance from the stir zone. Hence, on the advancing side and in the retreating side, after point X, there is a sharp increase in the hardness zone which is due to the increase in distance from the stir zone and also due to the partial cold work effect of T87 temper. Table 4 lists the hardness difference between the point marked point X and the adjacent point on the hardness map. The hardness difference of 38.1 VHN is observed for 900-90 joint due to faster cooling rate. This indicates that higher the rotational speed, higher the temperature generation in the advancing side, higher the hardness difference between the neighborhood points. Hence, this confirms the fact that intermetallic compounds (precipitate) formed is more in 900-90 joint than in 900-110 joint. It is also interesting to observe that the lower hardness plateau for 900-110 joint (Row 3 of Table 4), extends from 0 to 5 mm in the advancing side which is also indicated in the hardness map in Figure 4 as a small plateau region. This also confirms scarce precipitate formation in the aforementioned lower hardness plateau region.

**Figure 5:**
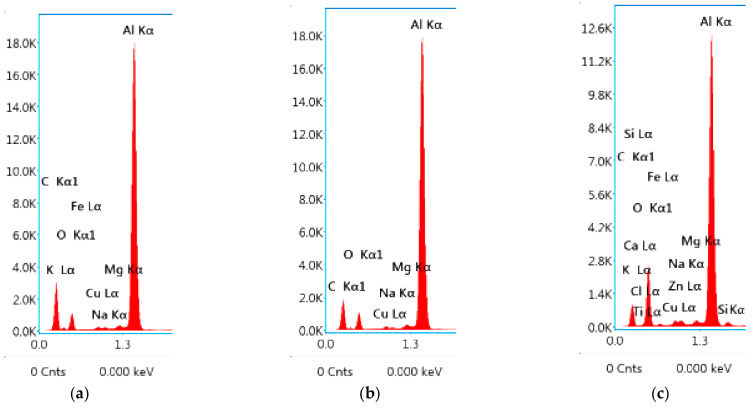
EDS spectrum analysis graph (**a**) Base metal (**b**) 900-90 joint (**c**) 900-110 joint.

**Figure 6:**
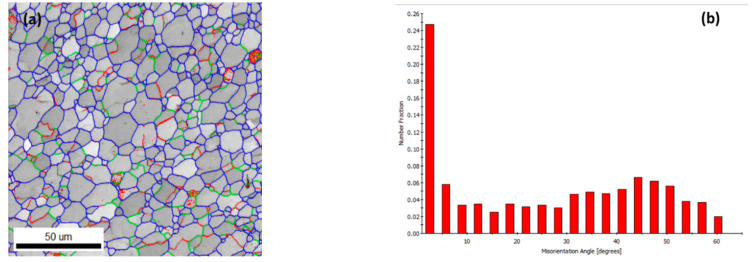

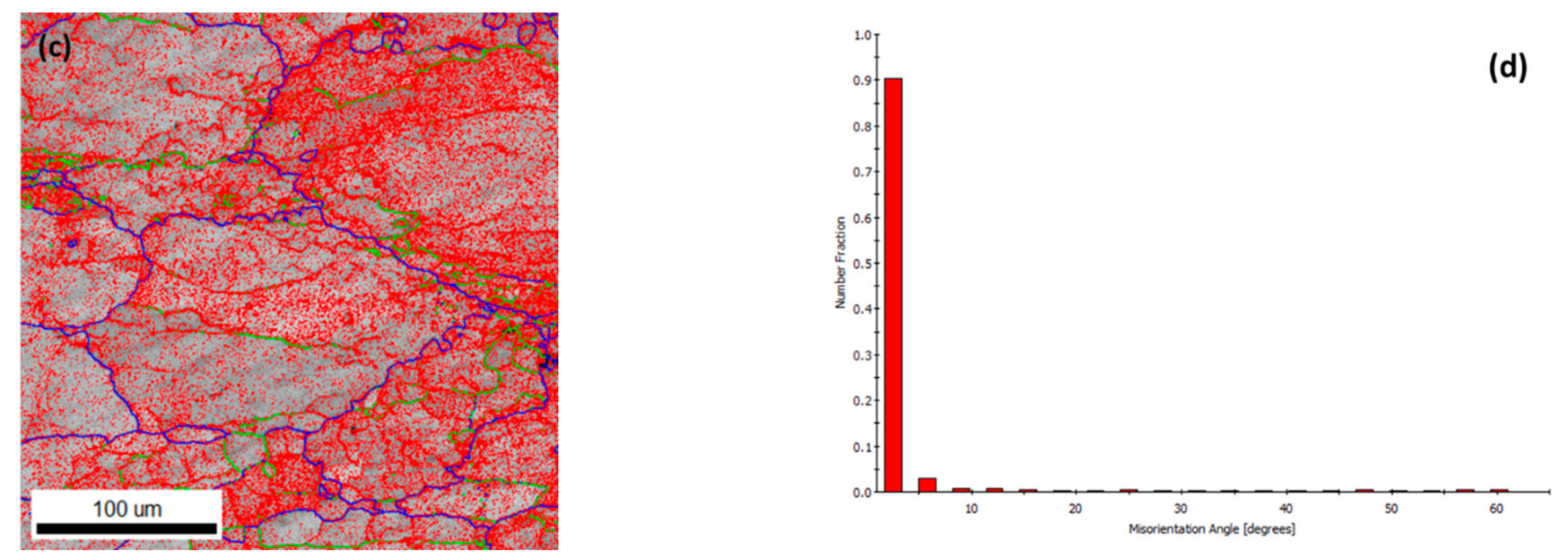
Grain Boundary maps for 900-90 joint (**a**) HAGB-LAGB map—S (**b**) Grain mis-orientation—SZ (**c**) HAGB-LAGB map—HAZ (**d**) Grain mis-orientation—HAZ.

It is observed from Figure 5a–c (EDS spectrum) and Table 5, that Aluminum (Al) is present in a major amount followed by Copper (Cu), Magnesium (Mg) and Iron (Fe) elements on both the base metal and in the weld joint. Fe formed the precipitate Al-Cu-Fe with Cu and Al, which created detrimental effect to the joint integrity in the grain boundary (eutectics) which is also clear from EDS spectrum. However, Cu and Mg formed Al-Cu-Mg precipitate escalated the joint integrity. Hence, the precipitate formation and grain boundary strengthening mechanisms assisted in the bond formation in the stir zone as shown in Figure 6a–d. Figure 6a,b shows the density of HAGB (High Angle Grain Boundary) and Low Angle Grain Boundary (LAGB) in the stir zone and the corresponding grain mis orientation. The HAGB is higher indicating the higher amount of stirring work in the weld zone and increased recrystallization. The grain boundary mis-orientation (>15°.) is observed in enormous amount which is the direct indication of recrystallized and rotated grains. The grain boundary strengthening will be carried out in the stir zone via accumulation of precipitates in the high angle and low angle grain boundary. Further, heat in the weld zone spreads to adjacent HAZ which increases the grain size and possibly precipitate size which is shown in Figure 6c,d. This increased grain boundary mis-orientation in the SZ dictates the fact that the orientation of the grains in the stir zone is not textured but randomized by the taper and threaded profile in the tool pin. The decreased grain mis-orientation in the HAZ dictates the fact that the zone experienced only heat without physical rotation. Particularly HAGB is very lesser in the HAZ. This phenomenon has decreased the hardness in the HAZ which is clearly shown in Figure 3 and Figure 4.

### 3.2. Corrosion Analysis

This section deals with the corrosion analysis of 900-90 joint and 900-110 joint. Corrosion analysis is a necessary test for this alloy as the application of this alloy is in aerospace and in airplane structural construction which operates in corrosive marine atmosphere.

**Figure 7:**
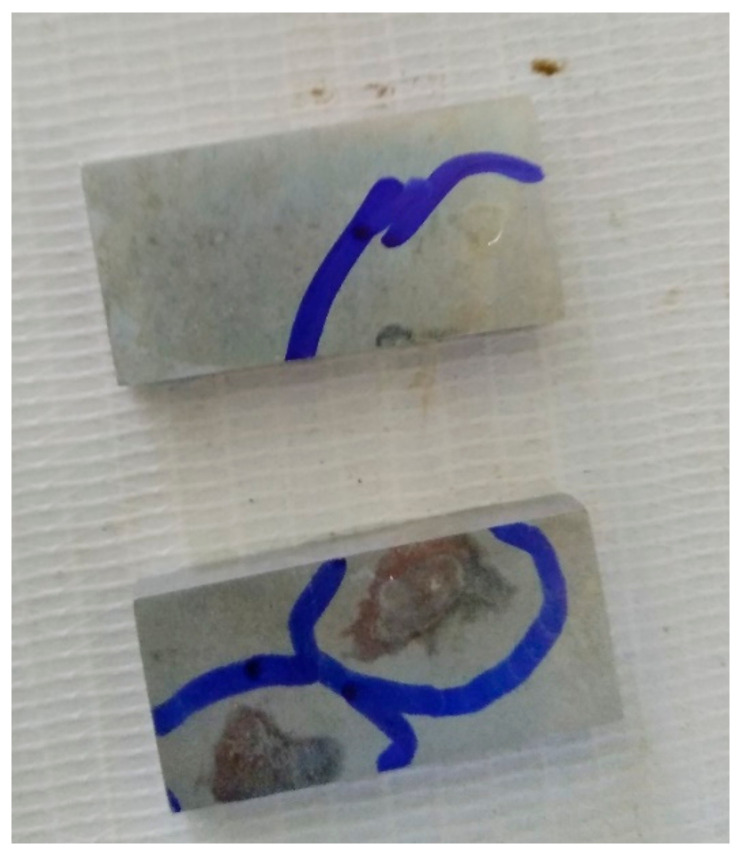
Corroded specimen after immersion test.

**Figure 8:**
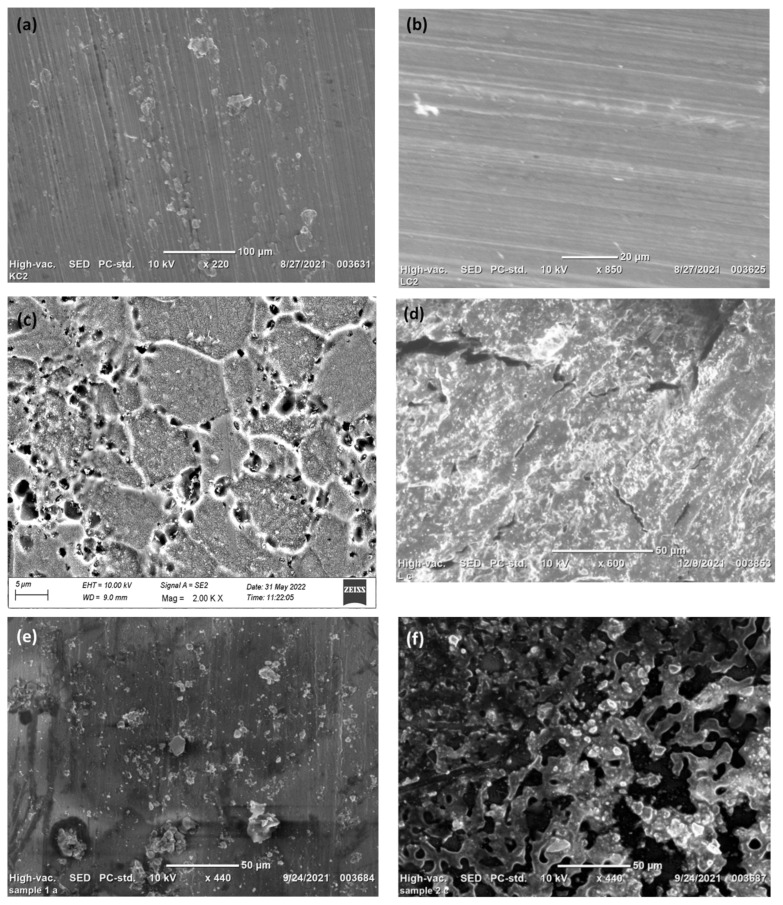

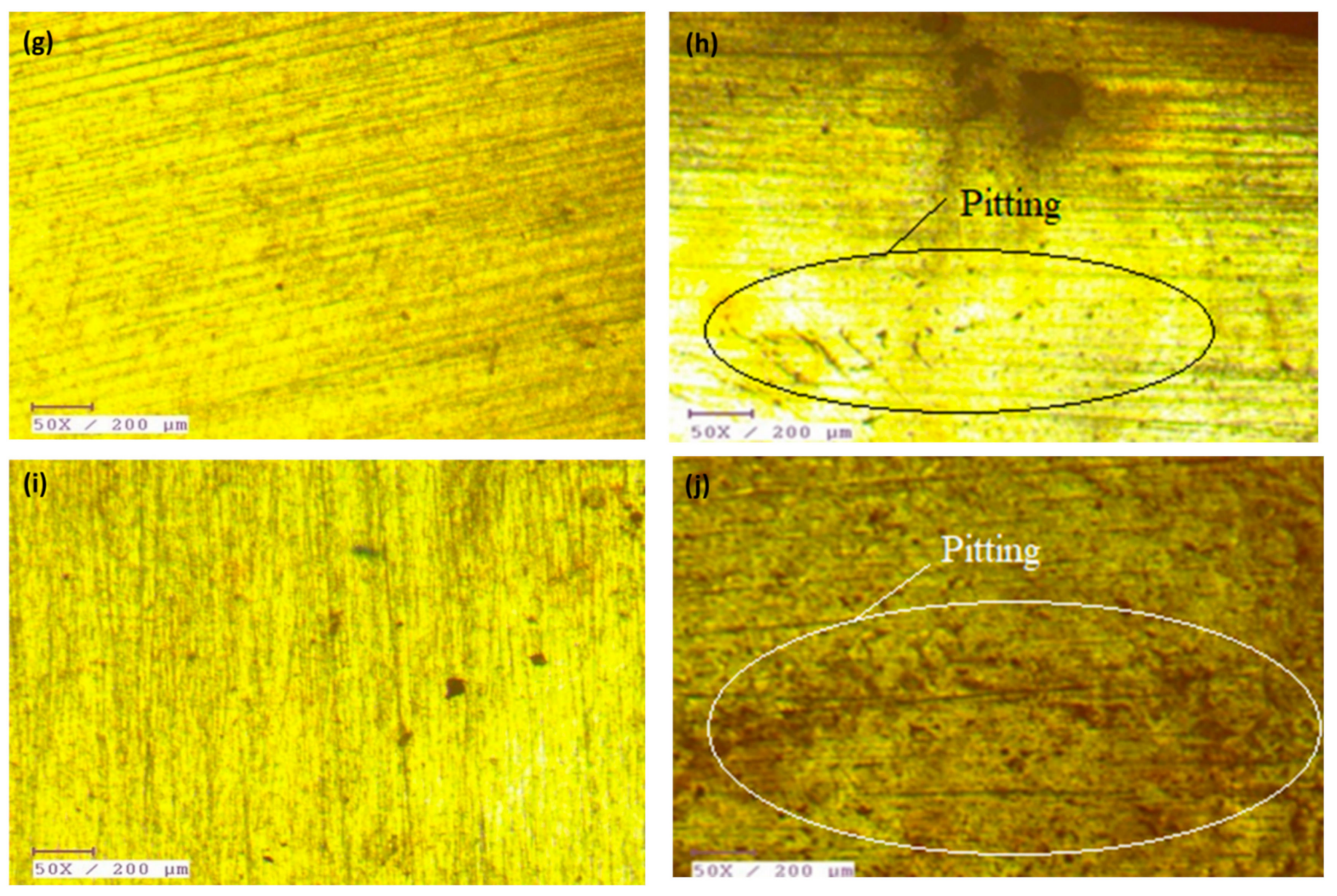
SEM micrographs (**a**) unetched 900-90 joint (**b**) unetched 900-110 joint (**c**) etched 900-90 joint (**d**) etched 900-110 joint (**e**) Corroded 900-90 joint (**f**) Corroded 900-110 joint (**g**) Optical microscopy image of 900-90 joint before corrosion (**h**) Optical microscopy image of 900-90 joint after corrosion (**i**) Optical microscopy image of 900-110 joint before corrosion (**j**) Optical microscopy image of 900-110 joint after corrosion.

Immersion corrosion test has been conducted by immersing the work in 57 g/L NaCl (catalyst) + 10 mL/L H_2_O_2_ (strong oxidizer) solution for 6 h at 32 °C to evaluate the corrosion behavior of the alloy. In the above said solution used for corrosion, NaCl is used as a catalyst used to accelerate the corrosive action between H_2_O_2_ and the metal. The corroded surface is shown in Figure 7 and is highlighted in the blue-colored area. Figure 8a–j indicates the SEM and optical microscopy images before and after corrosion. Figure 8a,b clearly indicates the track marks produced by the FSW tool and the induced shear stress (induced in the pin of the tool due to threads in the pin).

Figure 8 shows the intensified physical action of the tool on the work, and it displays the various microstructural deformities that occur during the process. Figure 8c,d indicates the sub surface morphology after etching is carried out. Figure 8c displays the grain surrounded by precipitates (Al-Cu) which is confirmed from EDS spectrum analysis (Figure 5). The precipitate (Al_2_Cu) formed on the grain boundaries are responsible for the hardness attained in the stir zone and in HAZ in 900-90 joint. Figure 8d shows the crack formed in the sub surface of the weld zone. Figure 8e,f presents pits formed in the weld zone after immersion corrosion test. These pits are the starting point of fracture on the surface. The fracture occurrence depends on the density of pits on the surface. The 900-90 joint shows lesser pits than 900-110 joint. This is due to the dissolution of precipitates in the stir zone in 900-90 joint due to heavy stirring. The higher number of precipitates formed in the 900-110 joint are responsible for the pits produced which is also confirmed from Figure 8g–j. Mass of the welded specimen before corrosion is 4.49 g. After corrosion, mass of the welded specimen is 4.479 g. for 900-90 joint. Hence, mass loss is 0.011 g. Mass of the welded specimen (900-110 Joint) before corrosion is 4.86 g. After corrosion, mass was reduced to 4.819 g. Mass loss is 0.041 g. This can be evidenced from the aforementioned SEM image. Hence, mass loss is more in the case of 900-110 joint. Cu wt.% in 900-110 joint is higher than in 900-90 joint which is evident from Table 6. This dictates the fact that Al_2_Cu (the predominant precipitate) corroded the aluminum phases more in 900-110 joint.

**Figure 9:**
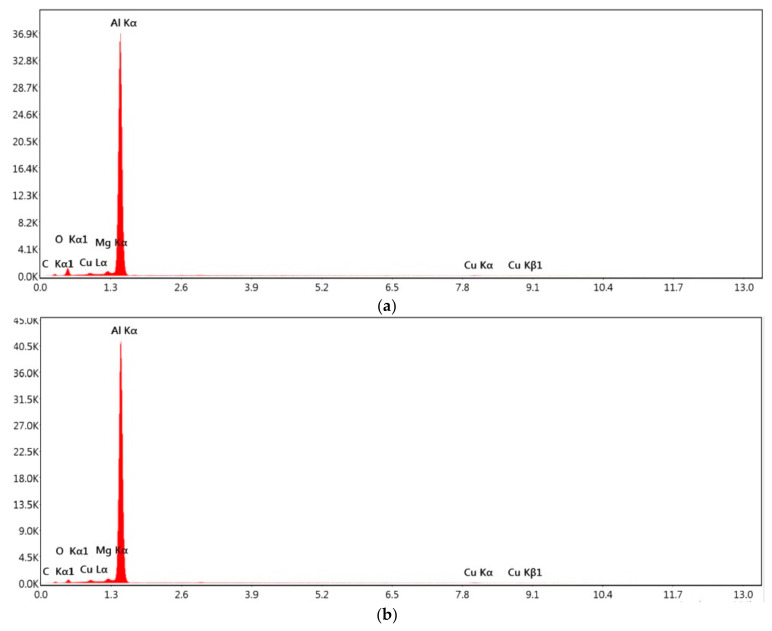
EDS spectrum analysis after immersion corrosion test (**a**) 900-90 Joint (**b**) 900-110 Joint.

From Figure 9, Table 6 and Table 7, it is understood that Cu and Mg co-exist in base metal and the same is reflected in the weld joint. Cu is the noble metal in corrosion perspective. Hence, Cu acts as cathode and Al matrix acts as anode. Hence, the aluminum matrix is corroded by the precipitate Al-Cu-Fe, Al-Cu (Table 5) and formed Cu and Mg rich phases (Table 7). Table 6 clearly shows wt.% of Cu present in the base metal. From Table 5 and Table 7, it is inferred that Cu is higher in wt.% in 900-110 joint than in 900-90 joint which led to more corrosive impact in 900-110 joint. Hence, the rate of corrosion is directly proportional to the wt.% Cu present in the joint and to the corrosive medium. Another interesting observation is considerable increment in Mg (1.1% to 2%) after corrosion which induces considerable corrosion resistance. The precipitate Al-Cu-Mg has been exposed by corrosion activity. One common aspect can be noted from the chemical composition (from EDS spectrum) before and after corrosion is the presence of Cu and Mg which contributed not only for corrosion but also to the enhancement of hardness in the weld joint.

## 4. X-ray Diffraction Analysis on Corroded Specimens

This section compares the X-ray diffraction analysis of 900-90 joint and 900-110 joint with that of base metal to appreciate the phase changes that happened after corrosion.

**Figure 10:**
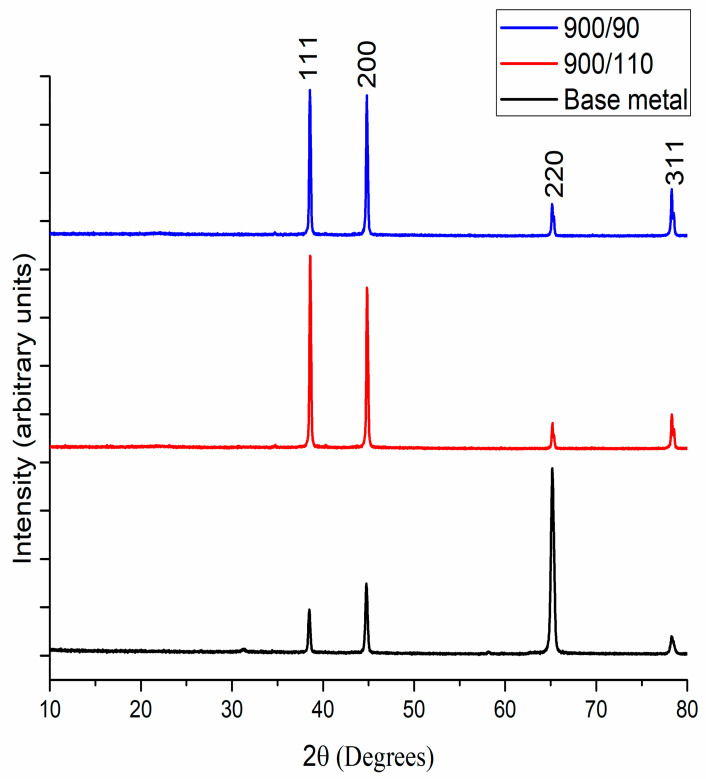
X-ray diffraction pattern of welded sample after corrosion analysis.

From Figure 10 of XRD analysis it is clear that all odd/even miller indices confirmed that the material analyzed has FCC structure. From EDS analysis and XRD analysis, the change in wt.% Al from 47.5% (uncorroded) to 84.9% (corroded) is reflected in the XRD peak of 900-90 joint and the change in wt.% Al from 42.5% (uncorroded) to 88.3% (corroded) is reflected in the XRD peak of 900-110 joint. The primary reason for the increase in the (111) peak is the evolution of Al(OH)_3_ which is responsible for the corrosion pit formation. Primary source of Al(OH)_3_ has evol3ved from H_2_O_2_ solution which has been used for the corrosion test. Secondary source of hydroxide is from dissolved oxygen in NaCl solution. Hence, these facts confirm that corrosion has occurred in the surface of the 900-90 and 900-110 joint which is also reflected in the SEM and optical microscopy analysis as previously mentioned.

### Effect of Corrosion on Surface Roughness and Hardness of the Joint

This section presents the influence of corrosion on change in surface roughness and hardness of the specimen. Surface roughness is one of the important properties to be analyzed in the perspective of the possibility of crack formation from the surface due to different forms of corrosion phenomena.

The surface roughness and hardness of the welded specimens before and after corrosion is analyzed and presented in Table 8. From the tabulated result, it is understood that the surface roughness values increased after corrosion. Hence, the presence of rougher surface is confirmed which is indicating the presence of small pits and also indicated the initiation of crack is possible from the surface. The hardness before the corrosion is 121.8 VHN for 900-90 joint and 98.3 VHN for 900-110 joint (Figure 3 and Figure 4). The hardness values after the corrosion are 125.2 VHN in the stir zone for 900-90 joint and 117.5 VHN in the stir zone for 900-110 joint. This hardness increase after the corrosion could be due to increase in Cu and Mg wt.% as indicated in Table 7.

## 5. Conclusions

In this work, mechanical properties such as hardness of the alloy, surface roughness and microstructure of the joint are analyzed before and after corrosion using various testing methods. The following observations have been made from the tests conducted:

- The recrystallization behavior, grain boundary strengthening and precipitate formation in the grain boundaries were observed in the stir zone of the weld joint.
- The hardness variation in the weld stir zone was analyzed before and after corrosion and minor change in the hardness is noticed after corrosion. The hardness before corrosion for the 900-90 joint is 73.3% of the base metal and 54.5% of the base metal for 900-110 joint. The hardness increase has been observed after corrosion which was 2.79% for 900-90 joint and 27.3% for 900-110. This hardness variation is the indication of evolution of Al-Cu precipitates in the grain and in the grain boundaries.
- Corrosion analysis has been performed and found that higher density of pits was formed in the 900-110 joint than in the 900-90 joint which is due to the presence of increased wt.% of Cu in 900-110 joint and hydroxides formed during corrosion mechanism. The mass loss % per year is 0.2% for 900-90 joint and 0.8% for 900-110 joint. This confirms the fact that more no. of Cu containing precipitates were formed in 900-110 joint.
- From EDS spectrum before corrosion and after corrosion, it was observed that the alloying elements Al, Cu, Mg, Fe contributed to corrosion behavior.
- Surface roughness analysis has been carried out to analyze the irregularity in the surface after corrosion and found that the surface roughness values escalated to 24.5% and 116% for 900-90 and 900-110 joints after corrosion.
- The traverse speed variation had led to severe corrosion which further led to the deviation in the surface roughness and also hardness of the weld joint. The underlying phenomenon for all these variations was precipitate formation and grain boundary strengthening.

## Figures and Tables

**Table 1:** Terminology used for welded plates.

Sl. No.	Rotating Speed	Traverse Speed	Description of Joint
1.	900 RPM	90 mm/min.	900-90
2.	900 RPM	110 mm/min.	900-110

**Table 2:** Chemical composition of AA8090 aluminum alloy.

Elements	Al	Li	Cu	Mg	Si	Zr	Cr	Mn	Ti
wt.%	95.2	2.35	1.29	0.88	0.04	0.11	0.0004	0.004	0.0.038

**Table 3:** Chemical composition of the tool material—H13 Tool steel.

Elements	Fe	Cr	Mo	Si	V	C	Ni	Cu	Mn
wt.%	90	5	1.7	1	1	0.37	0.3	0.25	0.4

**Table 4:** Hardness variation table in the vicinity of heat affected zone (HAZ).

Sl. No.	Description	Spacing mm	Side	Hardness Difference, VHN	Remarks	Reference
1.	Hardness profile for 900-90 joint	Between 10 mm and 15 mm	AS	38.1	The slope increases from 10 mm	Figure 3
2.	Hardness profile for 900-90 joint	Between 10 mm and 15 mm	RS	23.5	The slope increases from 10 mm	Figure 3
3.	Hardness profile for 900-110 joint	Between 15 mm and 20 mm	AS	28.2	The slope increases from 15 mm (Deviation observed)	Figure 4
4.	Hardness profile for 900-110 joint	Between 10 mm and 15 mm	RS	23.3	The slope increases from 10 mm.	Figure 4

AS—Advancing side RS—Retreating side.

**Table 5:** Chemical elements present in the base-metal and in the weld joint.

Sl. No.	Joint Description	Al (wt.%)	Cu (wt.%)	Mg (wt.%)	Fe (wt.%)
1.	Base metal	40.1	0.7	0.9	0.5
2.	900-90 Joint	47.1	0.9	1.1	-
3.	900-110 Joint	42.5	1.8	1.1	2.5

**Table 6:** Chemical elements in the weld joint after corrosion test.

Sl. No.	Joint Description	Al (wt.%)	Cu (wt.%)	Mg (wt.%)
1.	900-90	84.9	1.1	2.0
2.	900-110	88.3	1.5	2.0

**Table 7:** Influence of Cu on corrosion in the Base metal.

Alloy	Cu	Mg	Li	Cu/Mg
AA8090	1.4	0.8	2.3	1.75

**Table 8:** Surface roughness and Hardness before and after corrosion.

Sl. No.	Joint Desc.	R_a_ before Corrosion (µm)	R_a_ after Corrosion (µm)	Hardness before Corrosion (VHN)	Hardness after Corrosion (VHN)
1.	900-90	4.067	5.064	121.8	125.2
2.	900-110	0.566	1.223	92.3	117.5

## Data Availability

Not applicable.
